# Autoimmune encephalitis: frequency and prognosis

**DOI:** 10.1007/s00415-019-09273-5

**Published:** 2019-03-15

**Authors:** A. Al-Ansari, N. P. Robertson

**Affiliations:** 1 Department of Neurology, University Hospital Wales, Cardiff, NP14 4XN UK; 2 Division of Psychological Medicine and Clinical Neuroscience, Cardiff University, University Hospital of Wales, Heath Park, Cardiff, CF14 4XN UK

## Introduction

Progress in diagnostics and immunotherapy for autoimmune encephalitis has significantly improved survival rates. However, autoimmune encephalitis remains a challenging condition, associated with a protracted recovery course and uncertain long-term outcome. Clarification of the risk factors for autoimmune encephalitis, and the development of scales to aid with prognostication, may help improve clinical management.

This month’s journal club explores three papers relating to autoimmune encephalitis. The first paper examines the frequency of and risk factors for autoimmune encephalitis following herpes simplex encephalitis. The second paper describes the development of a score that predicts 1-year functional status in patients with anti-NMDA receptor encephalitis. The third paper discusses the development of a general scale for assessing severity in diverse autoimmune encephalitis syndromes.

## Frequency, symptoms, risk factors and outcomes of autoimmune encephalitis after herpes simplex encephalitis: a prospective observational study and retrospective analysis

It is well established that herpes simplex encephalitis can trigger autoimmune encephalitis. This paper explores the association between herpes simplex encephalitis and consequent autoimmune encephalitis using prospective (cohort A) and retrospective (cohort B) analyses. Cohort A comprised 51 patients with newly diagnosed herpes simplex encephalitis. These patients were followed up at several stages over a 12-month period with a neurological assessment and evaluation of serum and CSF autoantibody levels. Cohort B comprised 48 patients with a clinical diagnosis of autoimmune encephalitis following successful treatment of herpes simplex encephalitis.

In the prospective analysis, 14 patients (27%) developed neurological worsening consistent with probable autoimmune encephalitis. All 14 patients (100%) tested positive for IgG antibodies; 9 patients tested positive for anti-NMDA receptor antibodies, and 5 for other antigens. All patients had been negative for neuronal antibodies at onset of herpes simplex encephalitis. In contrast, of the 37 patients who did not develop clinical autoimmune encephalitis, 11 (30%) tested positive for IgG antibodies during follow up. The time period between herpes simplex encephalitis and the development of autoimmune encephalitis for cohort A patients ranged between 7 and 61 days. There were no statistically significant differences between clinical symptoms of herpes simplex encephalitis, cerebrospinal fluid parameters, radiological findings or treatment duration between patients who developed autoimmune encephalitis to those who did not.

In cohort B, 44/48 (92%) had neuronal antibodies either against NMDA (34/44) (77%) or against unknown antigens. Of all 58 patients who developed autoimmune encephalitis across both cohorts, patients younger than 4 years were more likely to present with choreoathetosis, whereas patients over age 4 years more frequently demonstrated behavioural problems and psychosis.

Comment: This is a robust study demonstrating a 27% probability of developing autoimmune encephalitis following herpes simplex encephalitis. Positive antibodies 3-week post-diagnosis of herpes simplex encephalitis may predict autoimmune encephalitis. Strengths include the objectivity of serum and CSF antibody testing and the follow-up of patients in the prospective analysis at a number of different time-points. However, it is unclear which autoantibodies, other than anti-NMDA, were tested for, and whether antibodies against unknown antigens found in the sera and CSF are genuinely pathogenic.

Armangue et al. (2018) Lancet Neurol 17:760–72.

## A score that predicts 1-year functional status in patients with anti-NMDA receptor encephalitis

The objective of this retrospective observational study was to construct a grading score that predicts neurological function 1 year after the diagnosis of NMDAR encephalitis.

This study examined a cohort of 382 patients identified as having anti-NMDA receptor encephalitis defined by the clinical picture as well as positive serum or CSF findings. All patient CSF and serum samples were sent either to the University of Pennsylvania or the University of Barcelona from 200 centres in 35 countries. All patients within the cohort had been part of a prior observational study, with previously collected data regarding demographics, symptom onset, clinical, laboratory and radiological features, time to treatment initiation, time from treatment initiation to improvement, and functional status using the modified Rankin scale (mRS). Good functional status at 1 year was defined as a mRS ≤ 2.

The group analysed a range of clinical factors to determine predictors of functional status at 1 year using a multivariate regression model. Five independent variables were identified including: need for ICU admission, delay of treatment initiation beyond 4 weeks of symptom onset, lack of clinical improvement 4 weeks after initiating treatment, presence of an abnormal MRI scan and CSF WBC count > 20 cells/uL. Points were assigned to each of these variables to construct the NEOS score (anti-NMDAr encephalitis 1 year functional status score), with a maximum possible score of 5. A strong association was found between the NEOS score and functional status at 1 year, with a NEOS score of 0 relating to good functional status and higher NEOS scores relating to greater disability (*p*$<$ 0.001).

Comment: The use of the NEOS score may aid with prognostication and identify a subset of patients who could benefit from treatment escalation. However, as the authors point out, 1 year functional status provides a snapshot in time, and ongoing recovery could be expected beyond this time-period. With regards to methodology, the pooling of patients from 35 different countries raises questions as to variations in treatment and the potential impact of this on clinical outcome.

Balu et al. (2019) Neurology Jan 15;92(3):e244–e252.

## Development of the clinical assessment scale in autoimmune encephalitis (CASE)

This study describes the development of a novel scale for assessing severity of disease and evaluating response to treatment in patients with diverse autoimmune encephalitis syndromes. Currently the modified Rankin Scale is used to assess neurological outcome in autoimmune encephalitis, however, as the mRS is designed to evaluate disability post stroke, a rating scale for autoimmune encephalitis is desirable.

Two expert panels developed the scale. Initially, 4 experts in autoimmune encephalitis generated the key clinical determinants of severity of autoimmune encephalitis. Thirteen neurologists then reviewed the key items put forward by the expert panel and answered whether the item was or was not essential for reviewing severity of autoimmune encephalitis. The final scale comprised nine items: seizure, memory dysfunction, psychiatric symptoms, consciousness, language problem, dyskinesia/dystonia, gait instability and ataxia, brainstem dysfunction and weakness, with sub-criteria for each key item giving a possible score of 0–3, and a maximum score of 27 for all nine key items. The scale was tested by three neurologists on a development cohort of 50 patients with confirmed autoimmune encephalitis, and by two neurologists on a validation cohort of 38 patients to evaluate its reliability. The scale showed excellent inter-observer and intra-observer reliability and good correlation with the modified Ranking Scale (*p* < 0.001).

Comment: CASE may help to overcome some of the current limitations in the assessment of autoimmune encephalitis. However, there are also a number of limitations to this study, in particular the key items within the scale were drawn up by small panel of neurologists via unclear means. Also the small number of assessors applying the scale to the development and validation cohorts makes it difficult to draw reliable inferences about inter-observer reliability, and the length of the scale with multiple subsections may also make its use cumbersome in clinical practice. Finally, the authors do not provide information as to how the scale ratings relate to overall outcome in autoimmune encephalitis patients.

Lim et al. (Jan 2019) Ann Neurol 85 (3):352–358.

## Conclusion

The first paper discussed raises awareness of the likelihood of autoimmune encephalitis following herpes simplex encephalitis. Clinicians should have a low threshold to test for autoimmune encephalitis in patients who deteriorate following viral encephalitis. The second and third papers demonstrate encouraging work in the development of assessment scales for autoimmune encephalitis. Although treatment and recovery remains an individualised process, these scales may help to identify patient subgroups who could benefit from novel therapies.

